# Machine Learning Models Using Post-operative CRP Trends to Predict Colorectal Anastomotic Leak: A Pilot Study

**DOI:** 10.7759/cureus.97423

**Published:** 2025-11-21

**Authors:** Hugo Woffenden, Zaid Yasen, Bhavika Rajesh, Alexander Hotouras

**Affiliations:** 1 General Surgery, Whipps Cross Hospital, London, GBR; 2 Trauma and Orthopaedics, Royal Free Hospital, London, GBR; 3 Surgery and Cancer, Imperial College London, London, GBR

**Keywords:** anastomotic leakage, colorectal surgery, c-reactive protein, extreme gradient boosting (xgboost), machine learning

## Abstract

Anastomotic leak (AL) remains a major cause of postoperative morbidity after colorectal resection. Postoperative C-reactive protein (CRP) thresholds have not been widely adopted as a predictive tool for AL, with recent studies assessing the predictive accuracy of the CRP trajectory. This pilot study evaluated machine learning (ML) models using postoperative CRP thresholds and trajectory data to predict AL, compared to conventional univariate CRP metrics.

A retrospective analysis of elective large bowel resections (2020-2025) from a single-institution database was performed. AL within the index admission or 30-day follow-up was diagnosed radiologically or intraoperatively. CRP levels from postoperative days (PODs) 1-3 were used to derive absolute values, percentage change, and net difference. Logistic regression and ML models - Lasso, Random Forest (RF), and Extreme Gradient Boosting (XGB) were trained with five-fold cross-validation. Metrics included area under the ROC curve (AUC), sensitivity, specificity, positive predictive value (PPV), and negative predictive value (NPV).

A total of 679 patients were included; AL occurred in 2.36% (16/679). XGB using absolute CRP values and percentage change demonstrated the highest performance (AUC 0.91, sensitivity 0.88, specificity 0.86, NPV 0.98). XGB modelling achieved comparable accuracy with absolute CRP values and net difference feature sets (AUC 0.90, sensitivity 0.81, specificity 0.93, NPV 0.97). Integrated threshold and trajectory feature set models outperformed exclusive absolute CRP value models (RF: AUC 0.84, sensitivity 0.69, specificity 0.92, NPV 0.95). Univariate logistic regression showed inferior discriminatory performance: POD 2 CRP ≥108 mg/L yielded AUC 0.87 (sensitivity 0.94, specificity 0.69, NPV 0.99), while POD 1→2 net increase ≥94 mg/L gave AUC 0.74 (sensitivity 0.56, specificity 0.94, NPV 0.94).

ML models integrating CRP threshold and trajectory data improved predictive accuracy for AL compared to traditional CRP thresholds. XGB and RF outperformed Lasso modelling, with balanced sensitivity and specificity. This highlights the value of dynamic, data-driven analysis in early postoperative risk stratification. Multi-centre analysis is planned for external validation and to confirm generalisability.

## Introduction

Anastomotic leak (AL) is a major complication following colorectal surgery with reported rates between 2-14% [1-4]. Early detection is crucial to reduce morbidity, mortality, and facilitate early and safe discharge. However, the diagnosis of AL remains between 6-15 days after surgery, reflecting the absence of a reliable diagnostic test to detect AL in the early postoperative course [5,6]. Delay in diagnosis is associated with prolonged hospital stay and increased mortality [7,8]. 

C-reactive protein (CRP) is a commonly used biomarker clinically to detect infectious complications postoperatively. Its validity as an early detector of AL has been widely investigated. A recent large meta-analysis demonstrated high discriminatory performance of CRP thresholds at postoperative day (POD) 3 with a sensitivity and specificity of 95% [9]. This exceeded previous meta-analyses' accuracies that concluded the reliability of CRP threshold exclusively as a negative predictor test, with POD CRP cut-offs of 148-172 mg/L reported [9-11]. Recent studies have investigated the CRP trajectory to further expedite AL diagnosis and improve model reliability by eliminating individual patient and surgical variations [12-14].

Machine learning (ML) offers the advantages over conventional threshold analysis by integrating non-linear interactions, temporal dynamics, and inter-patient variability. ML approaches have shown promise in postoperative AL risk prediction. Existing literature predominantly focuses on perioperative risk factors [15,16] or aims to combine postoperative serum and peritoneal biochemical markers [17] into a unified model. To our knowledge, no previous study has evaluated ML models incorporating postoperative CRP thresholds and trajectories to predict AL following large bowel resection.

This pilot study aimed to assess the predictive accuracy of ML combining CRP absolute and trajectory data. Secondary objectives were to compare model performance with univariate CRP thresholds and evaluate the balance between sensitivity and specificity. We hypothesised that ML approaches would demonstrate superior discrimination compared to conventional univariate CRP threshold, facilitating early postoperative risk stratification.

## Materials and methods

Study design and participants

A retrospective observational case-control study was conducted at a single NHS hospital trust between January 2020 and June 2025. All adult patients (≥18 years) undergoing elective colorectal resection with primary anastomosis (ileocolic, ileorectal, colo-colonic, colorectal or coloanal) for any indication (malignant, benign or IBD) were eligible. An AL was defined as radiological or operative evidence of a defect in the enteric wall at the site of the anastomosis. Patients managed surgically and conservatively were included. Exclusion criteria included: emergency procedures, defunctioning stoma formation without anastomosis, stoma reversal without resection, and preoperative sepsis. Cases with incomplete CRP data for POD 1-3 were excluded. No imputation was performed due to the time-series dependency of CRP features. The cohort was divided into an AL (n = 16) and a non-AL (n = 663) control group. From the control group, a subset of 152 patients was selected using a random sampling algorithm to mitigate computational imbalance for detailed CRP value analysis.

All clinical management, including investigation and treatment of AL and decisions regarding discharge readiness, was at the discretion of the treating teams. Use of mechanical bowel preparation and formation of diverting stomas was determined by individual surgeon preference. Clinicians were not blinded to postoperative CRP results. This study was approved by the institutional research ethics committee in April 2025 which waived the need for informed consent.

Data collection

Demographic, operative, and laboratory data were obtained from the institutional surgical database and electronic health records. Follow-up was completed within 30 days for all patients. Absolute CRP values were collected for POD 1-3, and from these, CRP trajectory metrics were derived - percentage change and net difference.

Statistical analysis

Univariate logistic regression was performed on each metric (absolute CRP, percentage change, and net difference) for consecutive POD intervals to assess baseline discriminatory ability. Multivariate models were developed using Lasso logistic regression, Random Forest (RF), and Extreme Gradient Boosting (XGB), trained across five feature sets: (a) CRP absolute values (PODs 1-3); (b) CRP percentage change (POD 1→2, 2→3, 1→3); (c) CRP net difference (POD 1→2, 2→3, 1→3); (d) CRP absolute values with CRP percentage change; (e) CRP absolute values with CRP net difference.

Data was randomly split into training and testing sets using five-fold cross-validation. Performance metrics included area under the receiver operating characteristic curve (AUC), sensitivity, specificity, positive predictive value (PPV), and negative predictive value (NPV) with a 95% confidence interval (CI).

Categorical data were presented as numbers with percentages and analysed using chi-square or Fisher’s exact test. Continuous variables are presented as mean (standard deviation) or median (interquartile range), depending on distribution. The t-test and Mann-Whitney U test were used for comparing parametric and non-parametric data, respectively. Delong’s test was used to compare model performance. A two-sided p-value of <0.05 was considered statistically significant.

Model implementation

ML models were implemented in Python 3.12.0 using scikit-learn 1.6.1, pandas 2.2.3, numpy 2.2.4, statsmodels 0.14.4, and XGBoost 3.0.0 libraries. Default parameters were used, with no additional hyperparameter tuning or early stopping performed. No imputation, outlier trimming, or feature scaling/normalisation was applied. Class imbalance was not explicitly adjusted. Stratified five-fold cross-validation with shuffling and a fixed random seed (42) was used to preserve AL/non-AL proportions in each fold. Pairwise AUC differences between models were evaluated using a z-test approximation to the DeLong method for correlated ROC curves (two-sided α = 0.05) applied to pooled out-of-fold predictions.

The Python code used for modelling is available at GitHub (https://github.com/HugoWoff/ML-Models-CRP-Anastomotic-Leak.git).

## Results

Characteristics of patients

During the study period, 679 patients were included in the primary analysis. Clinical and demographic characteristics of the groups are shown in Table 1. Overall, 16 patients (2.4%) developed AL. Median length of hospital stay was significantly longer in the AL group compared with the non-AL group (16 days (IQR 12-28) vs 6 days (IQR 5-9), p < 0.001). In addition, 3 (18.8%) patients were readmitted, and one patient (6.3%) died as a result of AL.

**Table 1:** Patient characteristics and management Note: Data are median (IQR), mean (SD) or n (%). Test statistic: Mann-Whitney U test (U), independent t-test (t), Fisher’s exact test (OR), chi-square test (χ²). Abbreviations: Charlson Comorbidity Index (CCI); The American Society of Anaesthesiologists (ASA); Body mass index (BMI).

Patient characteristics	Anastomotic leakage; n = 16 (2.4%)	No anastomotic leakage; n = 663 (97.6%)	p-value	Test statistic
Age (years)	54 (44-66)	59 (49-71)	0.34	U = 1038.5
BMI (kg/m^2^)	25.8 (4.3)	26.7 (4.5)	0.43	t = −0.792
Gender			0.44	OR = 1.588
Male	7 (41.2)	367 (55.4)		
Female	9 (56.2)	296 (44.6)		
CCI	3.38 (3.14)	4.04 (2.20)	0.22	U = 989.5
ASA			0.07	χ² = 5.423
I	1 (6.2)	13 (2.0)		
II	8 (50)	506 (76.3)		
III	7 (43.8)	144 (21.7)		
IV	0 (0.0)	0 (0.0)		
Surgical indication			<0.001	χ² = 17.712
Benign	6 (37.5)	52 (7.8)		
Malignant	7 (43.8)	567 (85.5)		
IBD	3 (18.8)	44 (6.6)		
Surgical technique			<0.001	χ² = 15.94
Open	9 (56.3)	100 (15.1)		
Laparoscopic	6 (37.5)	454 (68.5)		
Robotic	1 (6.3)	109 (16.4)		
Types of anastomoses			<0.001	χ² = 29.628
Ileocolic	6 (37.5)	271 (40.9)		
Ileorectal	1 (6.2)	14 (2.1)		
Colo-colonic	1 (6.2)	26 (3.9)		
Colorectal	6 (37.5)	352 (53.1)		
Coloanal	2 (12.5)	0 (0.0)		
Diverting ostomy	5 (31.2)	144 (21.7)	0.36	OR = 0.61

The median time to AL diagnosis was 7.0 days (IQR 5.0-10.0). No statistically significant differences were observed in age, BMI, sex distribution, or morbidity. Leak rates varied significantly based on indication for surgery (p<0.001), surgical technique (p<0.001) and level of anastomosis (p<0.001), with the AL group having a lower proportion of malignancies (AL 44% vs. non-AL 86%), a greater proportion of open surgeries (AL 56% vs. non-AL 15%) and more distal anastomoses overall. Construction of a diverting ileostomy did not interfere with the prevalence of AL (AL 31.2% vs non-AL 21.7%, p = 0.36). Of the 16 patients who had an AL, six (37.5%) underwent reoperation, two (12.5%) required radiological intervention (percutaneous drainage), and eight (50%) were managed conservatively with antibiotics.

Univariate analysis

As shown in Table 2, absolute CRP values demonstrated superior predictive power compared with percentage change or net difference. POD 2 absolute CRP achieved the highest discrimination (AUC 0.87) at an optimal cut-off of 108 mg/L, yielding high sensitivity (93.8%) but moderate specificity (69.4%). POD 3 CRP showed high discernment (AUC 0.83) with a cut-off of 184.4 mg/L, giving sensitivity of 75% and specificity of 91.9%.

**Table 2:** Diagnostic characteristics of absolute CRP values utilising univariate logistical regression analysis Performance metrics with a 95% CI. Abbreviations: postoperative day (POD); C-reactive protein (CRP); area under curve (AUC); positive predictive value (PPV); negative predictive value (NPV).

POD	Optimal Threshold (mg/L)	AUC	Sensitivity	Specificity	PPV	NPV
1	132	0.61 (0.44 -0.79)	0.44 (0.25-0.75)	0.92 (0.69-1.00)	0.44 (0.21-1.00)	0.92 (0.90-0.96)
2	108	0.87 (0.78-0.95)	0.94 (0.69-1.00)	0.69 (0.64-0.94)	0.31 (0.27-0.67)	0.99 (0.95-1.00)
3	184	0.83 (0.69-0.95)	0.75 (0.56-1.00)	0.92 (0.7-0.96)	0.57 (0.31-0.79)	0.96 (0.94-1.00)

Among the derived trajectory metrics (Table 3), the most accurate was the POD 1→2 net difference (AUC = 0.74, sensitivity 0.56, specificity 0.94) with an ideal cut-off of a 94 mg/L increase, though notably inferior to POD 2 and 3 absolute values. Similarly, percentage change showed its highest discrimination for POD 1→2 (AUC 0.63) at a cut-off of a 149% increase and was associated with a poor sensitivity-specificity balance (sensitivity 56%, specificity 88%).

**Table 3:** Diagnostic characteristics of CRP percentage change and net difference utilising univariate logistical regression analysis Performance metrics with a 95% CI. Abbreviations: postoperative day (POD); C-reactive protein (CRP); area under curve (AUC); positive predictive value (PPV); negative predictive value (NPV).

POD	Optimal Threshold	AUC	Sensitivity	Specificity	PPV	NPV
Δ% 1→2	+149%	0.63 (0.43-0.8)	0.56 (0.31-0.81)	0.88 (0.74-0.97)	0.41 (0.26-0.71)	0.93 (0.90-0.97)
Δ% 2→3	-13%	0.44 (0.32-0.57)	0.63 (0.00-1.00)	0.51 (0.22-1.00)	0.16 (0.00-0.21)	0.91 (0.87-1.00)
Δ% 1→3	+84%	0.60 (0.44-0.76)	0.38 (0.19-1.00)	0.91 (0.23-0.98)	0.38 (0.15-0.78)	0.91 (0.89-1.00)
Net Δ 1→2	+94	0.74 (0.58-0.90)	0.56 (0.38-0.88)	0.94 (0.71-0.99)	0.56 (0.25-0.86)	0.94 (0.91-0.98)
Net Δ 2→3	+85	0.40 (0.25-0.57)	0.56 (0.00-1.00)	0.45 (0.07-1.00)	0.13 (0.00-0.27)	0.88 (0.87-1.00)
Net Δ 1→3	+148	0.68 (0.51-0.84)	0.44 (0.25-0.94)	0.97 (0.34-1.00)	0.70 (0.17-1.00)	0.92 (0.90-0.98)

Multivariate analysis

Model performance for each feature set is summarised in Tables 4-8. Models with feature sets containing only one metric (CRP absolute values, percentage change, or net difference) over multiple days showed little or no improvement over their respective univariate predictors. ML models using only absolute CRP values achieved AUCs of 0.77-0.84, falling short of the logistic regression analysis for POD 2 absolute CRP (AUC 0.87) (Table 4). Similarly, utilising solely net difference achieved AUCs of 0.40-0.74, inferior to the highest value matching the POD 1→2 net difference univariate model (AUC 0.74) (Table 5). Models using only percentage change achieved modest but statistically significant gains in RF (AUC 0.79, p < 0.05) and XGB (AUC 0.77, p < 0.05) compared with the best univariate percentage change predictor (Δ% POD 1→2, AUC 0.63) (Table 6).

**Table 4:** Diagnostic characteristics of absolute CRP values utilising machine learning models Performance metrics with a 95% CI. Abbreviations: postoperative day (POD); C-reactive protein (CRP); area under curve (AUC); positive predictive value (PPV); negative predictive value (NPV); Random Forest (RF); Extreme Gradient Boosting (XGB)

Model	AUC	Sensitivity	Specificity	PPV	NPV
Lasso	0.77 (0.61-0.90)	0.75 (0.56-0.94)	0.83 (0.70-0.91)	0.39 (0.28-0.56)	0.96 (0.93-0.99)
RF	0.84 (0.70-0.96)	0.69 (0.56-1.00)	0.92 (0.67-0.99)	0.55 (0.27-0.93)	0.95 (0.94-1.00)
XGB	0.84 (0.67-0.97)	0.81 (0.56-1.00)	0.87 (0.82-0.99)	0.48 (0.38-0.93)	0.97 (0.94-1.00)

**Table 5:** Diagnostic characteristics of CRP net difference utilising machine learning models Performance metrics with a 95% CI. Abbreviations: postoperative day (POD); C-reactive protein (CRP); area under curve (AUC); positive predictive value (PPV); negative predictive value (NPV); Random Forest (RF); Extreme Gradient Boosting (XGB)

Model	AUC	Sensitivity	Specificity	PPV	NPV
Lasso	0.72 (0.56-0.88)	0.50 (0.38-0.94)	0.93 (0.55-0.98)	0.50 (0.21-0.83)	0.93 (0.91-0.99)
RF	0.74 (0.58-0.89)	0.69 (0.38-0.88)	0.81 (0.76-0.98)	0.34 (0.27-0.86)	0.95 (0.91-0.98)
XGB	0.74 (0.57-0.89)	0.56 (0.31-0.88)	0.94 (0.64-1.00)	0.56 (0.24-1.00)	0.94 (0.91-0.98)

**Table 6:** Diagnostic characteristics of CRP percentage change utilising machine learning models Performance metrics with a 95% CI. Abbreviations: postoperative day (POD); C-reactive protein (CRP); area under curve (AUC); positive predictive value (PPV); negative predictive value (NPV).

Model	AUC	Sensitivity	Specificity	PPV	NPV
Lasso	0.45 (0.31-0.61)	0.56 (0.00-1.00)	0.49 (0.04-1.00)	0.14 (0.00-0.20)	0.89 (0.87-1.00)
RF	0.79 (0.66-0.89)	0.88 (0.69-1.00)	0.69 (0.52-0.85)	0.29 (0.22-0.44)	0.97 (0.94-1.00)
XGB	0.77 (0.66-0.86)	0.94 (0.69-1.00)	0.55 (0.40-0.84)	0.23 (0.19-0.34)	0.98 (0.94-1.00)

**Figure 1:**
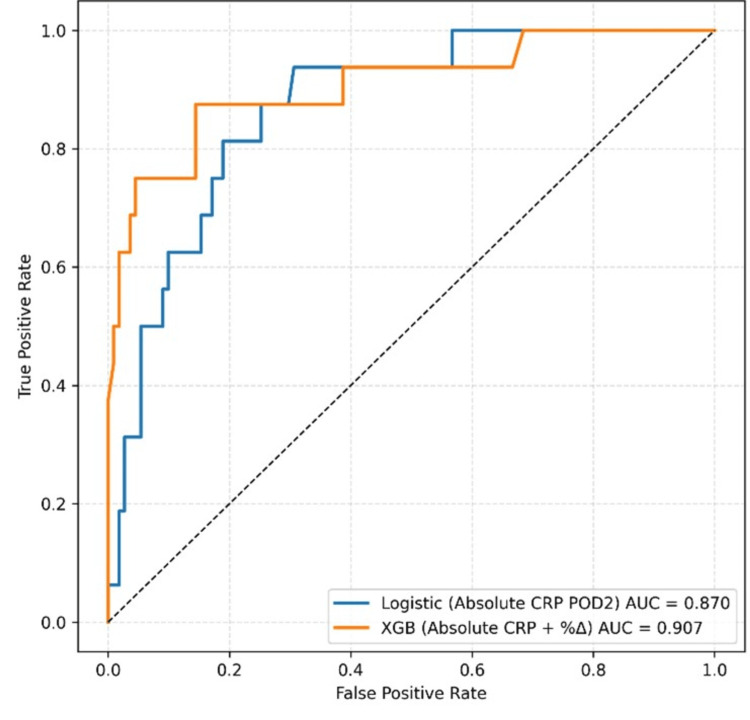
Receiver operator characteristic (ROC) curve comparing analysis of absolute CRP value POD 2 and Extreme Gradient Boosting (XGB) absolute CRP and percentage change (Δ%) models.

Combining absolute CRP with trajectory-based metrics (percentage change or net difference) consistently improved discrimination across all models. The highest-performing model was XGB trained on absolute CRP and percentage change (AUC 0.91, sensitivity 0.88, specificity 0.86) (Table 7). Comparable results were seen in RF modelling utilising the same feature set with unfavourable sensitivity-specificity trade-off (AUC = 0.90, sensitivity = 0.75, specificity = 0.96). XGB trained on absolute CRP and net difference performed similarly (AUC 0.90, sensitivity 0.81, specificity 0.93) (Table 8). All three top-performing models significantly outperformed univariate logistic regression, including POD 2 CRP (Figure 1), by DeLong’s test (p < 0.05).

Tree-based models (RF and XGB) consistently outperformed Lasso models trained on the same feature sets, which demonstrated variable performance (AUC range 0.45-0.77). Between the two models with first-tier performance, XGB showed optimal performance with selected feature sets combining absolute CRP values and trajectory metrics, percentage change (AUC: RF = 0.90, XGB = 0.91), and net difference (AUC: RF = 0.87, XGB = 0.90) (Tables 7-8).

**Table 7:** Diagnostic characteristics of absolute CRP values and percentage change utilising machine learning models Performance metrics with a 95% CI. Abbreviations: postoperative day (POD); C-reactive protein (CRP); area under curve (AUC); positive predictive value (PPV); negative predictive value (NPV); Random Forest (RF); Extreme Gradient Boosting (XGB)

Model	AUC	Sensitivity	Specificity	PPV	NPV
Lasso	0.79 (0.64-0.92)	0.69 (0.50-0.94)	0.89 (0.66-0.96)	0.48 (0.26-0.69)	0.95 (0.93-0.99)
RF	0.90 (0.78-0.98)	0.75 (0.63-1.00)	0.96 (0.64-0.99)	0.71 (0.28-0.94)	0.96 (0.95—1.00)
XGB	0.91 (0.80-0.98)	0.88 (0.63-1.00)	0.86 (0.80-0.99)	0.47 (0.39-0.93)	0.98 (0.95-1.00)

**Table 8:** Diagnostic characteristics of absolute CRP values and net difference utilising machine learning models Performance metrics with a 95% CI. Abbreviations: postoperative day (POD); C-reactive protein (CRP); area under curve (AUC); positive predictive value (PPV); negative predictive value (NPV); Random Forest (RF); Extreme Gradient Boosting (XGB)

Model	AUC	Sensitivity	Specificity	PPV	NPV
Lasso	0.77 (0.61-0.90)	0.75 (0.56-0.94)	0.83 (0.70-0.91)	0.39 (0.27-0.55)	0.96 (0.93-0.99)
RF	0.87 (0.74-0.98)	0.81 (0.63-1.00)	0.92 (0.78-0.98)	0.59 (0.36-0.87)	0.97 (0.95-1.00)
XGB	0.90 (0.78-0.98)	0.81 (0.69-1.00)	0.93 (0.76-0.98)	0.62 (0.36-0.86)	0.97 (0.96-1.00)

## Discussion

This single-centre pilot study demonstrates ML models integrating postoperative serum absolute CRP values with derived trajectory metrics achieved superior discriminatory performance compared with traditional univariate CRP absolute and trajectory metrics. This supports the hypothesis that ML can improve early prediction of AL following elective large bowel resection. The limitations of this study, notably the risk of model overfitting, limit the generalisability of these findings but support the development of a larger multicentre study for external validation.

Univariate threshold analysis

Our univariate findings are consistent with existing evidence that absolute CRP thresholds exhibit clinically useful discriminatory performance. This study demonstrated strong discriminatory power on POD 2 (AUC = 0.87, NPV = 0.99) and POD 3 (AUC = 0.83, NPV = 0.96), with POD 3 AUCs as high as 0.95 in meta-analysis [9]. POD 3 NPVs exceeding 0.95, as reported in this study and existing literature, are widely quoted as early safe discharge guides, but should be used with caution, given the relatively low incidence of AL in these studies [11,12,18,19]. This is reflected in POD 3 sensitivities ranging from 0.46-0.82 [11,12,18,19], in keeping with this study's findings of 0.75. This demonstrates a more modest but realistic ability of absolute CRP to rule out non-AL patients.

The unusually high POD 2 sensitivity (0.94) noted in this study may reflect exclusion of emergency colorectal resections and a study definition of AL limited to radiological or operative findings. Univariate threshold models typically prioritise specificity at the expense of sensitivity, limiting their reliability for early leak detection.

However, absolute CRP thresholds have some limitations. CRP production has been shown to vary between patients depending on patient factors, including BMI, as well as perioperative factors, including blood loss, ostomy formation, and duration of surgery [11,20,21]. Therefore, caution should be taken when generalising absolute CRP thresholds, and the potential benefits of the CRP trajectory considered as a moderator for patient and surgical factors.

Univariate trajectory analysis

Recent studies have investigated CRP trajectory metrics, percentage change, and net difference. In our cohort, both percentage change and net difference between PODs were less discriminatory than absolute CRP values when analysed in isolation. A POD 1-2 CRP net increase of 94mg/L demonstrated the greatest performance (AUC = 0.74), with a poor sensitivity (0.56) to specificity (0.94) trade-off and weak NPV (0.94) when accounting for the low AL incidence. CRP percentage change performed inferiorly, with the best discrimination seen between POD 1-2. A 149% increase was associated with a specificity of 0.88 but lacked sensitivity (0.56) and NPV (0.93).

This mirrors the results of Hoek et al., who found that a CRP increase greater than 50mg/L between PODs 1-3 lacked predictive value to singularly rule out AL [14]. Best performance between POD 1 and POD 3 showed a sensitivity of 0.65, specificity of 0.76, and negative predictive value of 0.94. In the large prospective multicentre PREDICT study, Stephensen et al. compared the predictive accuracy CRP trajectory and cut-off points over PODs 1-5 [12]. They concluded that trajectory testing, standardised at a CRP net increase >50 mg/L, provided improved specificity but lower sensitivity when compared with cut-off points. A change in CRP level of more than 50mg/l between POD 2 and 3 had a sensitivity of 0.32 with a specificity of 0.90 and an AUC of 0.61. In comparison, a POD 3 CRP cut-off yielded an AUC accuracy of 0.67 with a sensitivity of 0.46 and specificity of 0.74. They summarised that this made implicit sense given the nature of CRP modelling and that the concept of using both cut-off points and trajectory seemed intuitive. Our study concurs with current literature that trajectory measurements taken in isolation fail to show clear superiority over threshold cut-offs.

Modelling metrics over multiple PODs

Comparing metrics over multiple PODs is an alternate method of improving the diagnostic accuracy of CRP modelling in AL diagnosis. Stephensen et al. [12] and Neimann et al. [13] looked at trajectory measurements, net difference, and percentage change, respectively, over multiple PODs, demonstrating high sensitivity. The PREDICT study found a CRP increase of over 50mg/L between any two PODs from two to five increased sensitivity of 0.85 at the cost of specificity (0.51) when compared with isolated trend metrics. Similarly, Neimann et al. noted a 10% increase in CRP between any two consecutive days post POD 2, demonstrated robust modelling with a sensitivity of 1, a specificity of 0.83, and an NPV of 1.0 [13]. This model outperformed its CRP threshold comparators on the same data set, leading authors to conclude that trend modelling was superior to traditional CRP cut-offs.

This study found that absolute CRP values and net difference modelling utilising multiple PODs failed to demonstrate superior performance over their univariate counterparts. In RF and XGB modelling, utilising percentage change over all consecutive PODs significantly improved discriminatory ability as a result of increased sensitivity (Log = 0.56; RF = 0.88; XGB = 0.94) at the expense of loss of specificity (Log = 0.88; RF = 0.69; XGB = 0.55). These findings are an inherent product of the model design of utilising metrics from multiple PODs. The use of multiple data points expands the decision boundary, reducing the chance of missing an AL and increasing sensitivity whilst introducing potential noise in the data and exposing it to type I error and a drop in specificity. Therefore, this type of modelling lends itself to rule out AL and facilitate discharge as opposed to early diagnosis and expediting intervention.

Machine learning integration

Few studies have examined postoperative CRP in ML-driven AL prediction. Liu et al. combined perioperative immunonutritional indices (including postoperative CRP values) with ML algorithms, improving discrimination compared with absolute CRP values alone (AUC 0.89 vs 0.86) [15]. Sparreboom et al. analysed serum and peritoneal biomarkers, with multivariate penalised logistic regression modelling, achieving a C index of 0.78 - meaning 78% of the time the model assigned a higher probability to a patient with AL than a patient without AL [17]. Artificial Neural Networks were used by Adams and Papagrigoriadis when modelling pre- and postoperative clinical data [16]. Main predictors included CRP, platelet coun,t and preoperative hemoglobin,n achieving an AUC of 0.89, sensitivity of 0.85 and specificity of 0.82 for correct identification of clinical ALs.

To our knowledge, this study is unique in its use of ML models combining absolute CRP values with trajectory metrics. The highest performance outside these models was the logistic regression analysis of absolute CRP value on POD 2 with an AUC of 0.87, sensitivity of 0.94, and specificity of 0.69, thus forming the benchmark comparator. Highest overall discrimination was seen using XGB models with absolute CRP value and percentage change feature sets with an AUC of 0.91, sensitivity of 0.88, and specificity of 0.86. Tree-based combined feature set modelling produced a balanced sensitivity and specificity analysis that benefited from the addition of each metric. Absolute CRP feature sets produced high specificity with XGB achieving a sensitivity of 0.81 and specificity of 0.87. In contrast, trajectory measurements favoured high sensitivity models with XGB percentage changes, achieving a sensitivity of 0.94 and specificity of 0.69. The combination of static absolute CRP values with trajectory feature sets balanced the weaknesses inherent in each metric, suggesting that dynamic changes provide additive predictive value when contextualised by baseline CRP magnitude.

Superior discrimination was also seen when combining CRP absolute values with percentage change in RF (AUC = 0.90, sensitivity = 0.75, specificity 0.96) as well as when combined with net difference in the XGB model (AUC = 0.90, sensitivity = 0.81, specificity 0.93). Tree-based modelling consistently outperformed lasso regression utilising absolute CRP values percentage change (AUC = 0.79, sensitivity = 0.69, specificity = 0.89) or net difference (AUC = 0.77, sensitivity = 0.75, specificity = 0.83). This validates the tree-based model’s ability to more effectively capture non-linear relationships, including that of postoperative CRP metrics with AL risk.

Limitations

There are limitations to be addressed in this study. The low incidence of AL and small event rate introduces the risk of model overfitting despite cross-validation, as well as a class imbalance against the control. The single-centre, retrospective design, and lack of external validation restrict generalisability. These limitations will be addressed by the next phase of multicentre studies to validate our findings in a larger population.

This study did not include Brier scores or decision-curve analysis, which would have provided additional insight into model calibration and clinical utility. Future work will incorporate these to assess whether predicted probabilities are well-calibrated and whether the models confer net clinical benefit in decision-making contexts such as postoperative imaging or discharge planning.

Only elective operations were used in this study; thus, no conclusions can be made regarding the use of ML models in emergent operations. Furthermore, the definition of anastomotic leakage was based on operative or radiological findings. Patients with small ALs were potentially managed conservatively without radiological investigation, falling short of the diagnostic threshold outlined in this study. This may have resulted in the lower-than-expected leak rate, thus affecting sensitivity and PPV. Finally, we had no department-wide CRP protocol to guide collection or intervention based on results. Some patients had intermittent missing values and were discarded from the data analysis, biasing the sample.

## Conclusions

This pilot study showed that ML models improved internal performance in the early prediction of AL following elective large bowel resection. Combining absolute CRP values with trajectory metrics improved model accuracy with a balanced sensitive and specific performance. Tree-based models, particularly XGB, showed the greatest accuracy and support a paradigm shift toward dynamic, data-driven risk stratification in the postoperative setting. Despite the limitations in study design, these findings support the feasibility of ML models, with further external validation warranted. A multi-centre validation study is planned to confirm these findings in a larger cohort prior to clinical implementation.
